# The effectiveness of raising Hong Kong parents’ awareness of antimicrobial resistance through an education program with peer support on social media: a randomized, controlled pilot study

**DOI:** 10.1186/s12889-022-12697-w

**Published:** 2022-02-15

**Authors:** Pui-Lai Or, Tai-Yin Ching

**Affiliations:** 1 Department of Health and Physical Education, The Education University of Hong Kong, Hong Kong, China; 2 WHO Collaboration Centre for Infectious Disease Epidemiology and Control, School of Public Health, The University of Hong Kong, Hong Kong, China

## Abstract

**Background:**

The aim of this study was to address the misuse of antibiotics and test the feasibility of an education program with peer support on social media in improving parents’ knowledge on antimicrobial resistance at a regional level in Hong Kong. This pilot, if successful, will be developed into a main study.

**Methods:**

A cluster randomized controlled trial with two-arms were implemented. The intervention program consisted of two weekly sessions and each session lasted for 90 min. Parents in the intervention group would join a Facebook Page of Antibiotic Use, this online platform would allow participants to build a social network. A total of 48 parents had participated in the program. Parental knowledge and attitude were measured before and after the program using the Parental Perception on Antibiotics (PAPA) scale and the General Self-Efficacy Scale (GSE) to assess differences between and within the intervention and control groups.

**Results:**

All parents in the intervention group had an understanding that antibiotics could be effective at treating some infections only, as compared to 40% in the control group. All parents in the intervention group and 85% of the control group disagreed that they should reduce the dose of antibiotics when their children were recovering. The test was statistically significant (*p* = 0.039) at *p* < 0.05. There were a significant difference and a strong negative correlation between peer support on Facebook and the parents’ belief that antibiotics could be stopped when their children felt better, with Pearson coefficient of − 0.78 at *p* < 0.001. In general, there was no significant difference between the two groups with respect to the scale.

**Conclusions:**

Based on the findings in this pilot study, a further study focused on the education program with enhancement and peer support should be implemented on a larger scale with considerations of how it might support reducing incidence of antimicrobial resistance and potentially influencing prescription expectations of patients when seeking healthcare.

**Trial registration:**

Retrospectively registered Chinese Clinical Trial Registry ChiCTR2100044870.

Registered on 31 March 2021.

## Background

Overuse and misuse of antibiotics are the main causes of antimicrobial resistance which harm both individuals and the community [1]. The 2010 Eurobarometer and a 2017 study in Poland found that over 50% of adults still believed antibiotics could treat colds [2, 3]. A 2021 Malaysian survey found that majority of study respondents (67.5%) did not have adequate knowledge regarding antibiotics use and resistance [4]. A 2015 Hong Kong study found that 7.8% of the interviewees bought antibiotics without a prescription [5]. In a China study in 2020, one third (410) of the studied children had parental self-medication with antibiotics before medical consultation [6]. It is also evident that suboptimum compliance of antibiotic use, including taking leftover antibiotics from previous treatment courses and sharing unused antibiotics among household members or friends, is common in both developed and developing countries [7]. In particular, it has been found that antibiotics are commonly prescribed for children with medical conditions, including viral respiratory infections for which they provide no benefit [8]. Inappropriate use of antibiotics in children is a known issue and has generated widespread social concern [9]. Besides antimicrobial resistance, they can also lead to the development of adverse gastrointestinal effects in children [10]. It is not only parents but also parties such as antibiotic prescribers who contribute to the potential of antibiotic overuse. Many doctors report feeling pressurized by patients to prescribe antibiotics for viral infections such as influenza or cold [11]. To reduce antibiotic overuse in children, strategies need to be based on current knowledge and understanding such as knowledge in respiratory illnesses and their treatment [10]. Parents, as carers of their children and the primary medicine administrators, have a key role to play. However, they have inadequate knowledge about antibiotics and infectious diseases [10]. Some parents diagnose and medicate their children with antibiotics without consulting medical opinion first. In view of the potential for harm through this behavior, parents should be educated first with accurate information on both antibiotics and infectious diseases instead of having access to antibiotics without prescriptions.

In this study, we adopted a Behavior Change Technique Taxonomy Version 1 [12] to specify the intervention so as to induce parents’ positive behavior change in the health needs of their children. We hypothesized that with peer support this could motivate parents’ learning efforts and enhance their awareness of antibiotics resistance. Most education programs on antimicrobial resistance focus on healthcare professionals, particularly clinicians [13]. There are, however, no such programs for adults of the general population, particularly the parents of children aged 12 or younger.^6^ [14, 15], We conducted a pilot study in a small sample of kindergartens to examine the participating parents’ ability to comprehend and follow antibiotic usage information before and after an education program whilst receiving peer support through an online social platform to achieve behavior and attitude change in antibiotic use. Our objective was to test whether parents’ awareness of antimicrobial resistance could be improved through this program.

## Methods

### Design

A cluster randomized controlled trial with two-arms were implemented to assess the efficacy of behavior change in decisions on antibiotic use in relation to childhood infection.

### Setting

For confidentiality, computer generated numbers were used as codes to recruit kindergartens and parents of kindergarteners aged under 7, as clusters, to the intervention and control groups.

### Participants

The subjects were selected according to the inclusion criterion that they were parents of kindergarteners aged below 7. Kindergartens were selected from the same region to ensure that the demographics of the two groups were comparable. Randomization was implemented by putting the sealed codes for selection inside a paper bag. The sample was then drawn randomly by an individual who was not associated with the study for allocation concealment to prevent selection and confounding biases. The parents in the intervention group were asked to join an education program on antibiotic usage and a peer support group, whereas those in the control group were only given information leaflets on antibiotics from the Centre for Health Protection.

### Procedure

The intervention program consisted of two weekly sessions and each session lasted for 90 min. The same infection control nurse conducted all of the training during the intervention to maintain standardization and continuity of the information. The participating kindergartens sent training reminders to the parents 1 week before each session. Interventions addressing antibiotic use were administrated in two formats: a functional session (Week 1: the basic knowledge on viral and bacterial infections) and an interactive session (Week 2: case studies on consulting behavior, management planning and experience sharing). The parents in the intervention group, together with a pharmacist and an infection control nurse, would join a Facebook Page of Antibiotic 1. Facebook was the most favorite social media platform, 72.4% of Hong Kong population use Facebook [16]. Other potential options were YouTube, Instagram and Twitter. With the features provided by the Facebook, consultation services would be provided via the social network by sending response to query. Qualified professionals from the team would answer these queries. The online platform would post news about antibiotic use or antimicrobial resistance twice weekly, and allow interactions among users, thus strengthening parent-to-parent support.

To facilitate evaluation of the participating parents, assessment packets containing two questionnaires and reminders to parents were sent to the participating kindergartens for dispatch to the intervention and control groups for use before the 2-week period. One participating parent of each child was asked to complete one set of questionnaires before and immediately after the program. The participants were instructed to fill out the questionnaires only once irrespective of the number of children attending the same kindergarten. The questionnaires took 20 min to complete.

The ethical approval for the study was obtained from the Human Research Ethics Committee of The Education University of Hong Kong (REF 2018-19-0122), and participants have provided written consent before taking part in the study. The parents were informed that withdrawal at any time would not result in any negative consequences. All data were protected with passwords. Only the researcher and her team had access to the datasets to prevent any leakage of sensitive information.

### Measures

To measure peer support, the peer support outcome protocol adapted from the outcome evaluation indicators [11] was used. Specific outcomes that are available in the protocol include: demographics, service use, program satisfaction, and participation in a discussion group.

Parental knowledge and attitude were measured pre- and post-program using the Parental Perception on Antibiotics (PAPA) scale and the Generalized Self-Efficacy scale (GSE) scale to assess differences, if any, between and within the intervention and control groups.

The PAPA scale was administered to assess the participants’ pre- and post-capability in comprehending and acting on antibiotics-related information (functional: the basic skills in understanding antimicrobial drug knowledge), with Cronbach’s alpha of 0.78. The PAPA scale consists of four sections: 1) Children’s health, 2) Antibiotics and health information, 3) Experience with antibiotics and health professionals, and 4) Personal attitudes and beliefs about antibiotics. It has 32 items measuring the factors influencing the overuse of antibiotics in children, especially those with upper respiratory tract infections. Parents were asked to rate on a 5-point Likert scale ranging from “strongly disagree” to “strongly agree” or from “never” to “always” on child health-related history, including the number of cold episodes and antibiotics (courses) used for the youngest child during the previous year (ranging from “never” to “more than 6 times” a year). Parents were asked if any of their children had contracted any serious infectious or chronic disease. There were also items asking about factors influencing antibiotics resistance and parental use of antibiotics including knowledge and beliefs, behaviors, adherence, information seeking, and awareness, and their perception about doctors’ prescribing behavior [8]. Parents in the control group were only given information leaflets on antibiotics from the Centre for Health Protection. The parents in both groups were required to complete a self-reporting GSE scale questionnaire at pre- and post-intervention. GSE scale is a 10-item measure with a score ranging from 1 to 4 each. Higher scores indicate stronger parental belief in self-efficacy. The Cronbach’s alpha coefficient for the entire scale was 0.80 and the test-retest reliability coefficient was 0.69 [13].

### Statistical analysis

Descriptive statistics were used to summarize characteristics of the data to provide information about the intervention and control groups of the participating parents selected from the 12 kindergartens.

Four statistical tests were used in this study. Chi-square tests were undertaken to ascertain differences in baseline characteristics within and across the intervention and control groups. Mann Whitney U tests were conducted to evaluate the significance of differences of the participants’ knowledge and attitude towards antibiotic use between the intervention and control groups. In case of insufficient significant differences on items of interest across the groups for the Mann Whitney U tests, to supplement the deficiency, Wilcoxon Signed Rank Tests would be run instead for the intervention group at the baseline (pre-intervention) and after the training (post- intervention) and differences between these observations would be analyzed for statistical significance. Finally correlation analysis was performed to determine the significance of the relationship between the social support and parents’ personal attitude towards antibiotic use. For the estimation of effects, 95% confidence intervals were used. The statistical significance for all tests was set at *p* < 0.05.

## Results

For the demographic characteristics, a total of 48 parents participated in the program with four parents dropping out from the control group because of sickness or personal issues. As a result, 24 parents participated in the intervention group and 20 in the control group. The sex distribution of the participants was 3 (12.5%) males and 21 (87.5%) females for the intervention group, and 3 (15%) males and 17 (85%) females for the control group. The age range was 22 to 43 and 21 to 45 for the intervention and control groups respectively. In the intervention group, the education background of 20 participants (83.3%) was graduation from secondary school and 4 (16.7%) from university, while in the control group, 19 (79.2%) from secondary school and 5 (20.8%) from university. No significant demographic differences were found between the two groups (Table 1).

**Table 1:** Demographic data of study participants

	Intervention group (*n* = 24), n (%)	Control group (*n* = 20), n (%)	P (< 0.05)
Parents’ characteristics			
Sex			
Male	3 (12.5)	3 (15)	0.06
Female	21 (87.5)	17 (85)	
Age, years	30.5 (12.5)	31.5 (10.8)	0.19
Education			
Secondary	20 (83.3)	17 (85.0)	0.53
University	4 (16.7)	3 (15.0)	0.21

When using Mann Whitney U tests to compare responses to items in Section 1 of the PAPA scale which covered children’s health records, before the intervention over half of the parents reported that the number of colds their youngest children had in the past year was 2 to 3 episodes for the intervention group and 3 to 4 for the control group. To treat the common cold, nearly half of the parents in both groups said that their youngest children had taken antibiotics 1 year before either once a year or 2 to 3 times for the intervention and control groups respectively. To prevent common cold, all parents in the intervention group and 30% in the control group responded that their children received seasonal influenza vaccine in the past 6 months. After the training, all parents in the intervention group disagreed that antibiotics are effective to fight against infections (virus, bacteria and fungi), as compared to 40% in the control group. From the Mann Whitney U test performed for the questions, a significant difference (*p* = 0.024) (Table 2) was found for Item 5 only. Also only Item 1 in Table 3 was found to be significantly different (*p* = 0.039) that all parents in the intervention group and 85% of the control group disagreed that they should reduce the dose of antibiotics if their children were recovering.

**Table 2:** Parents’ knowledge on proper antibiotic use with PAPA scale – comparison of intervention versus control group

Item		Strongly disagree (%)		Disagree (%)		Neutral (%)		Agree (%)		Strongly agree (%)		*P*-value
		Intervention (*n* = 24)	Control (*n* = 20)	Intervention (*n* = 24)	Control (*n* = 20)	Intervention (*n* = 24)	Control (*n* = 20)	Intervention (*n* = 24)	Control (*n* = 20)	Intervention (*n* = 24)	Control (*n* = 20)	
1.	Common cold: need to take antibiotics	75	35	25	55	0	10	0	0	0	0	0.142
2.	Sore throat: need to take antibiotics	25	25	0	45	50	30	0	0	25	0	0.053
3.	Antibiotics are used to treat bacterial infections	0	0	25	10	0	25	50	50	25	15	0.770
4.	Antibiotics are used to treat viral infections	50	10	25	10	0	30	0	50	25	0	0.222
5.	Antibiotics can cure all types of infections (viruses, bacteria and fungi)	50	10	50	30	0	40	0	20	0	0	0.024*
6.	Antibiotics help treat children with colds	50	10	25	30	25	30	0	30	0	0	0.077
7.	Antibiotics can be harmful to human health	0	0	0	15	50	40	25	40	25	5	0.481
8.	Some bacteria are getting harder to use antibiotics	0	0	0	10	0	10	50	65	50	15	0.107
9.	Some bacteria can become resistant to antibiotics if the dose is insufficient	0	0	0	0	0	30	50	45	50	25	0.183

**Table 3:** Parents’ experience with antibiotics with PAPA scale - comparison of intervention versus control group

Item		Strongly disagree (%)		Disagree (%)		Neutral (%)		Agree (%)		Strongly agree (%)		*P*-value
		Intervention (*n* = 24)	Control (*n* = 20)	Intervention (*n* = 24)	Control (*n* = 20)	Intervention (*n* = 24)	Control (*n* = 20)	Intervention (*n* = 24)	Control (*n* = 20)	Intervention (*n* = 24)	Control (*n* = 20)	
1.	If my child’s condition feels better, I can reduce the dose of antibiotics.	75	20	25	65	0	5	0	5	0	5	0.039*
2.	If my child is mild, I will give appropriate antibiotics according to the condition.	75	25	25	55	0	0	0	15	0	5	0.063
3.	There is no difference in skipping the dose of one or two antibiotics.	75	35	25	55	0	5	0	5	0	0	0.142
4.	If your child has symptoms of cough, cold or flu without taking antibiotics, my child will not get sick for a long time.	50	15	25	45	25	30	0	10	0	0	0.235
5.	If my child has a cold or cough, it is best to use antibiotics to cure	50	20	25	50	25	25	0	5	0	0	0.384
6.	When a child has a cold, using antibiotics can speed up healing	25	20	50	25	25	25	0	30	0	0	0.280
7.	Strict adherence to antibiotic dose is not important	75	35	25	50	0	10	0	5	0	0	0.137

A notable observation, though not statistically significant to draw any firm conclusions, was that Item 9 in Table 2 of the PAPA scale which asked whether parents agreed that some bacteria could become resistant to antibiotics if the dose was insufficient, 25% of the parents in the intervention group chose “neutral” (neither agree nor disagree) before the program and 100% of them chose either “agree” or “strongly agree” after the program. In contrast, the control group had 30% of the parents chose “neutral” to this item both before and after the 2 weeks.

From the pre- and post-assessments of the effects of the education program, it was found that out of the 9 items on parents’ knowledge on proper antibiotics use, 5 had significant Wilcoxon Signed Rank test *p* value of less than 0.05. Particularly for Item 4 where “Antibiotics are used to treat viral infections” before the training, only 8% of parents chose “strongly disagree” while after the training nearly half (44%) of the parents chose “strongly disagree” (Table 4). For the parents’ experience with antibiotics, all items had Wilcoxon Signed Rank test *p* value less than 0.05, which showed that there was statistically significant difference in experience and attitudes on the use of antibiotics. For Item 1 (“If my child’s condition feels better, I can reduce the dose of antibiotics.”), before the training only 14% of the parents strongly disagreed while after the training, more than half (53%) of the parents chose “strongly disagree” (Table 5).

**Table 4:** Parents’ knowledge on proper antibiotic use with PAPA scale - comparison of baseline and post intervention

Item		Strongly disagree (%)		Disagree (%)		Neutral (%)		Agree (%)		Strongly agree (%)		*P* -value
		Pretest (*n* = 24)	Posttest (*n* = 24)	Pretest (*n* = 24)	Posttest (*n* = 24)	Pretest (*n* = 24)	Posttest (*n* = 24)	Pretest (*n* = 24)	Posttest (*n* = 24)	Pretest (*n* = 24)	Posttest (*n* = 24)	
1.	Common cold: need to take antibiotics	20	40	17	29	33	15	27	12	3	4	0.001*
2.	Sore throat: need to take antibiotics	6	20	14	22	50	36	20	14	10	8	0.008*
3.	Antibiotics are used to treat bacterial infections	0	3	0	16	20	5	50	42	30	25	0.238
4.	Antibiotics are used to treat viral infections	8	44	3	27	14	13	66	10	9	6	0.001*
5.	Antibiotics can cure all types of infections (viruses, bacteria and fungi)	11	30	14	40	42	21	11	4	22	5	0.001*
6.	Antibiotics help treat children with colds	8	35	22	49	22	8	39	8	9	0	0.001*
7.	Antibiotics can be harmful to human health	8	8	6	8	42	33	36	40	8	11	0.399
8.	Some bacteria are getting harder to use antibiotics	14	0	3	0	8	19	70	70	5	11	0.122
9.	Some bacteria can become resistant to antibiotics if the dose is insufficient	14	10	3	0	3	9	53	47	27	34	0.957

**Table 5:** Parents’ experience with antibiotics with PAPA scale - comparison of baseline and post intervention

Item		Strongly disagree (%)		Disagree (%)		Neutral (%)		Agree (%)		Strongly agree (%)		*P*-value
		Pretest	Posttest	Pretest	Posttest	Pretest	Posttest	Pretest	Posttest	Pretest	Posttest	
		(*n* = 24)	(*n* = 24)	(*n* = 24)	(*n* = 24)	(*n* = 24)	(*n* = 24)	(*n* = 24)	(*n* = 24)	(*n* = 24)	(*n* = 24)	
1.	If my child’s condition feels better, I can reduce the dose of antibiotics.	14	53	20	34	16	0	27	13	23	0	0.001*
2.	If my child is mild, I will give appropriate antibiotics according to the condition.	8	38	30	41	14	5	39	10	9	6	0.002*
3.	There is no difference in skipping the dose of one or two antibiotics.	22	52	55	35	16	5	7	8	0	0	0.014
4.	If your child has symptoms of cough, cold or flu without taking antibiotics, my child will not get sick for a long time.	16	7	47	24	22	39	15	30	0	0	0.016
5.	If my child has a cold or cough, it is best to use antibiotics to cure	11	41	20	44	39	11	16	4	14	0	0.001*
6.	When a child has a cold, using antibiotics can speed up healing	8	35	16	38	16	13	53	14	7	0	0.001*
7.	Strict adherence to antibiotic dose is not important	5	30	28	27	25	16	28	27	14	0	0.004*

As for the effect of the program on the parents’ self-efficacy, no significant differences were found. Following pre- and post-assessments of the intervention group, Item 7 of Table 8 was found to be marginally significant.

Regarding peer support on social media, the goals of creating a Facebook support group on Antibiotic Use was in agreement with the data obtained from the PAPA scale items in that more than half of the parents obtained health-related information from the Internet. Through the Facebook Audience Insight Tool, the support group’s behavior on Facebook was tracked. The creation of the group was a success and platform was visited more than a hundred times a week with activities such as posting messages and comments, and interacting with peers about antibiotic use. Using regression analysis, a significant strong negative correlation was found between social support and parents’ belief that antibiotics could cure their children’s cold symptoms, with Pearson coefficient of − 1 and *p* = 0.001, implying that they had learnt that antibiotics are not useful for treating colds. In addition, there was a significant difference and a strong negative correlation between peer support in social media and the parents’ belief that antibiotics could be stopped if their children felt better after the intervention, with Pearson coefficient of − 0.78 and *p* = 0.001, implying that the program could change the parents’ belief on proper antibiotic use (Table 6).

**Table 6:** Parents’ source of health-related information and its correlation matrix

Item		Mean	SD	1	2	3
1.	I get health-related information from my family or friends.	3.40	0.598	1.000		
2.	I get health related information from the internet	3.50	0.688	0.128	1.000	
3.	I get health-related information from the social media.	3.45	0.887	0.139	0.733**	1.000

**p < 0.005

Although there was no significant difference between the two groups with respect to the GSE scale (Table 7), yet no adverse outcomes were captured in this study. One notable observation is the response to Item 7 in Table 8, which asked if it was true that the parents could remain calm when facing difficulties because I can rely on my coping abilities. It was found that selecting the “moderately true” option had increased from 33 to 63% after the program and this item was found to be significantly different (*p* = 0.050).

**Table 7:** Parents’ self-efficacy using General Self-efficacy (GSE) scale – comparison of intervention versus control group

Item		Not at all true (%)		Hardly true (%)		Moderately true (%)		Exactly true (%)		*P*- value
		Intervention (*n* = 24)	Control (*n* = 20)	Intervention (*n* = 24)	Control (*n* = 20)	Intervention (*n* = 24)	Control (*n* = 20)	Intervention (*n* = 24)	Control (*n* = 20)	
1.	I can always manage to solve difficult problems if I try hard enough	0	5	25	35	50	50	25	10	0.421
2.	If someone opposes me, I can find the means and ways to get what I want	0	10	50	60	50	25	0	5	0.429
3.	It is easy for me to stick to my aims and accomplish my goals	0	0	50	35	50	65	0	0	0.580
4.	I am confident that I could deal efficiently with unexpected events	0	5	50	35	50	55	0	5	0.727
5.	Thanks to my resourcefulness, I know how to handle unforeseen situations	0	20	50	35	50	40	0	5	0.678
6.	I can solve most problems if I invest the necessary effort	0	0	25	15	75	65	0	20	0.353
7.	I can remain calm when facing difficulties because I can rely on my coping abilities	0	10	25	25	75	65	0	0	0.641
8.	When I am confronted with a problem, I can usually find several solutions	0	5	50	20	50	70	0	5	0.351
9.	If I am in trouble, I can usually think of a solution	0	0	50	25	50	65	0	10	0.277
10.	I can usually handle whatever comes my way	0	5	50	35	50	45	0	15	0.614

**Table 8:** Parents’self-efficacy using General Self-Efficacy (GSE) scale – comparison of baseline and post intervention

Item		Not at all true (%)		Hardly true (%)		Moderately true (%)		Exactly true (%)		P-value
		Pretest (*n* = 24)	Posttest (*n* = 24)	Pretest (*n* = 24)	Posttest (*n* = 24)	Pretest (*n* = 24)	Posttest (*n* = 24)	Pretest (*n* = 24)	Posttest (*n* = 24)	
1.	I can always manage to solve difficult problems if I try hard enough	0	0	38	31	50	48	12	21	0.421
2.	If someone opposes me, I can find the means and ways to get what I want	8	8	63	46	29	42	0	4	0.429
3.	It is easy for me to stick to my aims and accomplish my goals	8	0	25	41	63	59	4	0	0.580
4.	I am confident that I could deal efficiently with unexpected events	4	2	30	34	59	52	7	12	0.727
5.	Thanks to my resourcefulness, I know how to handle unforeseen situations	11	4	24	50	59	42	5	4	0.678
6.	I can solve most problems if I invest the necessary effort	13	0	0	18	63	63	24	19	0.353
7.	I can remain calm when facing difficulties because I can rely on my coping abilities	0	0	50	19	33	63	17	17	0.050*
8.	When I am confronted with a problem, I can usually find several solutions	0	0	25	21	63	64	12	15	0.351
9.	If I am in trouble, I can usually think of a solution	4	0	30	21	58	60	8	19	0.277
10.	I can usually handle whatever comes my way	13	0	25	39	58	47	4	14	0.614

## Discussion

The aim of this pilot study was to test its feasibility of an education program with peer support on social media in improving parents’ knowledge on antimicrobial resistance at a regional level in Hong Kong. With a small sample size of 44 in this pilot, its analytical power is expected to be low and its predictions may be biased. However, we hope that this pilot can provide insights for the main study.

As stated by Item 1 in Table 3, it was found that the participants after the intervention group gained a better understanding that common colds do not need antibiotics. Although this finding there does not seem to be enough evidence that the program had a significant impact on correcting the misconception of using antibiotics for curing colds, this is acceptable for a pilot study and can be corrected by having a large enough sample size in the main study. Another support for the main study is the positive change in the response to Item 5 in Table 2; in that the participants’ knowledge of using antibiotics for all types of infections (viruses, bacteria, fungi) had a significant improvement for the intervention group over the control group. This result was similar to that of Ekambi et al.’s study [17]. Another finding revealed by Item 9 in Table 2 is that the knowledge on bacteria becoming more resistant to antibiotics was similar for both groups. This may provide insights to the modification of the education intervention program in the main study. Item 9 in Table 2 states that insufficient dose of antibiotics being the cause of antimicrobial resistance, the intervention group performed better than the control group. This finding coincides with the hypothesis of this study. From the collected data, both groups could not differentiate viral, bacterial and fungal infections, nor understand the effect of skipping antibiotics dosage in a medication course, and were not sure about whether antibiotics help speed up healing colds. This information about the participants is vital for the effectiveness of the intervention education program in the main study.

From the above observations, it is clear that parents learnt the basic knowledge on proper and inappropriate antibiotic uses with respect to antimicrobial resistance through an education programme. The education program, however, should be enhanced in view of the difficulties the parents had in answering questions related to cold and cough symptoms. This study supported the findings that family and friends influence medicine taking [18]. Moreover, this study also demonstrated that parents’ behavior was influenced by social support [19]. The findings demonstrated that parents who were active in the social media were able to learn correct information on antibiotics in ways that worked for them [20].

Parents’ perception of their self-efficacy affected their behavior. Although our findings showed only marginal significance, yet it can be observed that pre- and post-assessments showed improved parental self-efficacy in the intervention group while parental self-efficacy decreased in the control group. This result was similar to that of Gross et al. [21]. which found a trend of growing parental self-efficacy in their parent training groups as compared to those in the control groups though the difference was not statistically significant. It is necessary to increase parental self-efficacy to support the development of knowledge and communication skills on antibiotic use [22, 23] because one study had shown that parents with low self-efficacy were not able to put parenting knowledge into practice [19].

Limitations did exist with this study. First, the samples were small and they were selected from one region in Hong Kong. Second, it only investigated the parents of kindergarteners. We suggest further studies can be expanded to parents of primary school students. Third, it investigated the peer support on one social media platform - Facebook - only, we suggest further studies can include other social media to enrich existing literature. Fourth, despite no adverse outcomes observed on the social media platform, it shall be worthwhile to look for more advanced tools to track discussion contents to avoid misinformation being disseminated when the study is on a larger scale. Furthermore, we suggest to include parents’ feedback about the intervention and social media interaction in future study.

## Conclusion

This pilot yielded positive preliminary results on improving basic knowledge of antibiotic use to reduce antimicrobial resistance. It also demonstrates that peer support could increase the self-efficacy of parents to enhance their learning in these medication issues. Based on the findings in this pilot study, a further study focused on the education program with enhancement and peer support can be considered at a larger scale with considerations of how it might support reducing incidence of antimicrobial resistance. Moreover, expectation on reducing antimicrobial resistance which is unlikely to be possible except longitudinal comparison study carried out in future study.

## Data Availability

Derived data supporting the findings of the study is available from the corresponding author on request.

## Abbreviations

PAPA: Parental Perception on Antibiotics scale
GSE: General Self-Efficacy scale
